# Vanillin Activates HuTGA1-HuNPR1/5-1 Signaling to Enhance Postharvest Pitaya Resistance to Soft Rot

**DOI:** 10.3390/foods15010153

**Published:** 2026-01-03

**Authors:** Jian Xu, Xinlin Liu, Yilin He, Jinhe Li, Muhammad Muzammal Aslam, Rui Li, Wen Li

**Affiliations:** 1 Key Laboratory for Quality Regulation of Tropical Horticultural Crops of Hainan Province, School of Tropical Agriculture and Forestry, Hainan University, Danzhou 571737, China; x99321@126.com (J.X.); lxl021025@163.com (X.L.); starfox2024@163.com (Y.H.); lijinhe9@163.com (J.L.); liwen9-210@163.com (W.L.); 2 School of Breeding and Multiplication (Sanya Institute of Breeding and Multiplication), Hainan University, Sanya 572025, China; 3 Institute of Forestry and Pomology, Beijing Academy of Agriculture and Forestry Sciences, Beijing 100093, China; 2011ag3575@uaf.edu.pk

**Keywords:** pitaya, vanillin, disease resistance, inducing resistance, TGA transcription factor

## Abstract

*Fusarium oxysporum*-induced soft rot severely threatens postharvest pitaya quality and storage life, and while vanillin shows promise in the disease management, its mechanisms for controlling pitaya decay remain incompletely understood. In this study, we systematically investigated the molecular mechanism by which vanillin inhibits soft rot in postharvest pitaya, employing physiological and biochemical characterization, bioinformatics analysis, and molecular biology techniques. Compared with control fruit on 10 d, vanillin treatment significantly reduced disease index and lesion area by 27.12% and 67.43%, respectively. Meanwhile, vanillin treatment delayed the degradation of total soluble solids (TSSs) and titratable acidity (TA) and promoted the accumulation of total phenolics and flavonoids. Additionally, vanillin enhanced the activities of defense-related enzymes, such as catalase (CAT), superoxide dismutase (SOD), phenylalanine ammonia-lyase (PAL), *β*-1,3-glucanase (GLU), chitinase (CHI), peroxidase (POD) and polyphenol oxidase (PPO), and increased antioxidant capacity, as evidenced by increased DPPH radical scavenging capacity and ascorbic acid content. This resulted in reduced oxidative damage, as indicated by decreased levels of malondialdehyde (MDA), H_2_O_2_ and O_2_^•−^. Yeast one-hybrid (Y1H), dual-luciferase reporter (DLR) and subcellular localization revealed that HuTGA1, a nuclear-localized transcriptional activator, specifically bound to the as-1 cis-acting element and activated expression of *HuNPR1* and *HuNPR5-1*. Transient overexpression of *HuTGA1* reduced reactive oxygen species (ROS) accumulation and upregulated related genes. These findings suggest that vanillin treatment might enhance pitaya resistance by activating the HuTGA1-HuNPR signaling module, providing insights into the molecular mechanisms underlying vanillin-induced resistance.

## 1. Introduction

Pitaya (*Hylocereus undatus*), a non-climacteric fruit native to Mexico and Central and South America, has gained global recognition for its vibrant appearance, unique flavor, and high economic value. Its cultivation has expanded to tropical and subtropical regions, including Vietnam and China [1]. However, pitaya production faces significant challenges from biotic and abiotic stresses, leading to substantial postharvest losses due to fruit decay. Soft rot, caused by *Fusarium oxysporum* (*F. oxysporum*), is a particularly devastating disease that threatens the sustainability and growth of the pitaya industry [2]. Therefore, developing effective and safe preservation strategies is essential to maintaining fruit quality and maximizing economic returns.

Various postharvest preservation techniques have been explored to maintain pitaya quality, including chemical treatments, controlled atmosphere storage, coating treatments, and cold storage [3,4,5]. However, these methods have limitations, including potential environmental and health risks associated with chemical fungicides, accumulation of coating materials in the human body, and sensory quality impairment. Additionally, cold storage is energy-intensive, and controlled atmosphere preservation requires specialized equipment [6,7,8]. These limitations highlight the need for green, safe, and sustainable alternatives [9]. Consequently, research focus has shifted towards exploring natural, pollution-free preservation strategies that can effectively maintain pitaya quality while minimizing environmental and health risks.

Exogenous vanillin application has been shown to effectively delay quality deterioration in various horticultural products. Vanillin exhibits broad-spectrum antifungal activity against pathogens such as *Alternaria alternata* in apples and *Botrytis cinerea* in tomatoes. Its mechanism of action involves disrupting cell membrane integrity, inducing lipid peroxidation, and modulating defense-related enzymes. In apples, vanillin activates enzymes such as phenylalanine ammonia-lyase (PAL), *β*-1,3-glucanase (GLU) and Chitinase (CHI), inhibiting pathogen growth and improving fruit quality [10]. Similarly, in tomatoes, vanillin inhibits mycelial growth and spore germination, suppresses pathogen-related enzyme activities, and delays postharvest disease onset [11].

Vanillin-based treatments have shown promise in extending the storage life of various fruits. For example, chitosan–vanillin coatings have been reported to suppress *F. oxysporum* fruit rot in tomato by inhibiting pathogen growth and inducing defense-related enzymes, ultimately reducing disease development and extending postharvest shelf life [12]. Similarly, in grapes, the combined application of vanillin with clove and lavender essential oils encapsulated in chitosan or alginate microcapsules effectively inhibited the mycelial growth and spore germination of *Botrytis cinerea*, preserved fruit firmness and intrinsic flavor attributes, and consequently improved the overall postharvest quality [13]. The efficacy of these treatments is attributed to the inhibition of pathogen growth, induction of defense-related enzymes, accumulation of antifungal metabolites, and maintenance of cellular homeostasis, revealing vanillin’s potential as a natural preservative for postharvest disease control.

Salicylic acid (SA) signaling pathway plays a crucial role in plant defense against pathogens, involving the translocation of NPR1 protein and its interaction with TGA transcription factors to activate pathogenesis-related (PR) genes [14]. TGA transcription factors, which belong to the basic leucine zipper (bZIP) family, are important regulators of plant growth, development, and responses to biotic and abiotic stresses [15]. Accumulating evidence indicates that TGA transcription factors play critical roles in plant disease resistance, although their functions vary depending on the plant species and pathogen. In banana, *MaTGA8* enhances resistance to *F. oxysporum* through its interaction with *MaNPR1* and *MaNPR4* [16]. Similarly, *ScTGA4* of sugarcane contributes to resistance against *Xanthomonas albilineans* by regulating the salicylic acid (SA) signaling pathway [17]. In potato, *StTGA39* positively regulates the expression of *StBAK1*, thereby conferring resistance to bacterial wilt [18]. In grapevine, *VvTGA8* mediates resistance to white rot by modulating SA pathway–related genes, including *SlPR1* and *SlPR2* [19]. In contrast, certain TGA transcription factors, such as *CmTGA7* and *CmTGA9*, function as negative regulators and reduce resistance to Botrytis cinerea [15]. These findings highlight the complex and dual roles of TGA transcription factors in plant–pathogen interactions. However, their functional mechanisms in pitaya remain unclear and require further investigation. Vanillin, a naturally derived compound, exhibits both antimicrobial and resistance-inducing properties, making it a promising antagonist for postharvest disease control. It not only suppresses pathogen growth but also activates immune responses in fruits [15,20]. The molecular mechanisms of vanillin-enhanced resistance in pitaya, particularly the roles of the SA signaling pathway and TGA transcription factors, require further elucidation.

This study aims to explore vanillin’s role in enhancing pitaya resistance to *Fusarium*-induced soft rot, focusing on the regulatory function of *HuTGA1*. This study assessed vanillin’s effects on lesion expansion, antioxidant defense, antifungal metabolite accumulation, and PR gene expression, and validated *HuTGA1*’s role in fruit defense responses, providing insights into vanillin-induced resistance and sustainable disease management in tropical fruits.

## 2. Materials and Methods

### 2.1. Pitaya Fruit

Fresh pitaya fruit of “Jindu No. 1” were harvested at commercial maturity stage, corresponding to approximately 80% ripeness, with a predominantly or fully red pericarp and persistently green bracts. Fruits were meticulously selected following strict criteria, including uniform size, freedom from pest infestations and fungal/bacterial diseases, as well as the absence of physical damage. The selected fruits were subjected to a surface sterilization treatment using a 0.1% sodium hypochlorite solution, followed by air-drying at room temperature. The pathogen used in this study was isolated by our research group from naturally infected pitaya fruit, following the method described by Ge et al. [21]. *F. oxysporum* (1 × 10^6^ CFU mL^−1^) was cultured on PDA medium for 7 d and subsequently tested for pathogenicity on pitaya fruits. Vanillin (C_8_H_8_O_3_) of analytical grade was obtained from Macklin (204-456-2, Guangzhou, China). Approximately 200 pitaya fruits were randomly divided into two groups for further experimentation.

Pitaya fruits were immersed in vanillin (4 g L^−1^, 20 min) and sterile water (control), air-dried, and wound-inoculated with *F. oxysporum* (2 × 10^6^ CFU mL^−1^; 25 µL at three epidermal sites using a sterile toothpick) as described by Ge et al. [21]. Fruits (six per unsealed polyethylene bag, 0.02 mm; 20 × 30 cm) were stored at 25 ± 0.5 °C and 90% RH for up to 10 d; disease symptoms and lesion area were recorded to 10 dpi. Samples were taken every 2 d; 2-cm-wide peel and pulp strips from the equatorial region were excised, snap-frozen in liquid nitrogen, and stored at −80 °C for analysis. All assays used three biological replicates, each of five fruits [22].

### 2.2. Determination of Fruit Quality Attributes

Postharvest decay was assessed at 0, 2, 4, 6, 8, and 10 d by counting fruits with visible symptoms; disease severity was scored using a six-level decay index (DI) based on the percentage of decayed area [23], and lesion area was calculated as S = 3.14 × (lesion diameter/2)^2^ from the mean of nine diameters [24]. Weight loss was determined on six fruits, calculated using the formula: weight loss (%) = (1 − M2/M1) × 100 with initial weight M1 and subsequent weights M2 recorded every 2 d [5,25].

Total soluble solids (TSS), titratable acidity (TA) and AsA were determined using 1 g of fresh fruit pulp for each analysis. TSS content was determined using a digital handheld refractometer (PAL-1, ATAGO, Tokyo, Japan). Three replicates were analyzed for each treatment group. TA was determined by titration with 0.1% NaOH to pH 7.5 using phenolphthalein (P991927, Macklin, Shanghai, China) as an indicator, after homogenization with 4 mL of deionized water, and expressed as % citric acid [25]. Ascorbic acid (AsA) was quantified by 2,6-dichlorophenolindophenol titration using 4 mL of 2% oxalic acid as the extraction solvent and expressed as mg 100 g^−1^ FW [26].

Malondialdehyde (MDA), total phenols (TP) and flavonoids (TF) were determined using 0.5 g of fresh fruit pulp for each analysis. MDA was assayed by the TBA method using 2.0 mL of 0.67% TBA solution, and measured at 450/532/600 nm, MDA content was expressed as μmol g^−1^ FW [27]. Total phenolic compounds and flavonoids were determined following the procedure described by Yang et al. [28] with slight modifications. Samples were extracted with 5 mL of 80% methanol, and the resulting extracts were used for analysis. TP content was measured at 760 nm using gallic acid as the standard, while TF content was determined at 510 nm using rutin as the standard. Results were expressed as mg g^−1^ FW.

### 2.3. Determination of Reactive Oxygen Species (ROS) Level and Defense-Related Enzyme Activities

ROS and antioxidant/defense enzymes were assayed from frozen peel. The rate of O_2_^•−^ generation was measured following Tang et al. [27] using 0.5 g tissue homogenized in ice-cold phosphate buffer (1 mM EDTA, 0.3% Triton X-100, 2% PVP) and expressed as μmol min^−1^ g^−1^ FW; H_2_O_2_ was quantified using 0.5 g of peel tissue with a commercial kit (Nanjing Jiancheng Bioengineering Institute Co., Ltd., Nanjing, China) (Nanjing Jiancheng) against a standard curve. A 0.5 g of peel tissue was mixed with 2 mL of 0.2 mM DPPH dissolved in ethanol, and the absorbance was measured at 517 nm [29]. DPPH radical-scavenging activity was calculated as (%) = (1 − Sample absorbance/Control absorbance) × 100.

Crude enzyme extracts (0.5 g of peel tissue ground in liquid nitrogen) were used for the determination of the following enzyme activities. Catalase (CAT) activity was measured by monitoring H_2_O_2_ decomposition at 240 nm, and superoxide dismutase (SOD) activity was determined based on the inhibition of nitro blue tetrazolium (NBT) photoreduction at 560 nm, both enzyme activities were determined according to the method described by Yun et al. [30], using an extraction buffer containing 3.0 mL of 100 mmol/L phosphate buffer (pH 7.5) with 5 mmol/L DTT and 5% PVP. Peroxidase (POD) activity was determined in a 3.0 mL reaction system containing 50 mmol/L acetate buffer (pH 5.5), using guaiacol as the substrate and recording absorbance at 470 nm, while polyphenol oxidase (PPO) activity was assessed in the same buffer system, with catechol as the substrate and absorbance detected at 410 nm [22]. PAL was measured at 290 nm using L-phenylalanine in 3 mL of 50 mM borate buffer, pH 8.8 [31]. GLU activity was determined using laminarin as substrate and measuring absorbance at 540 nm after the DNS color reaction, while CHI activity was measured with colloidal chitin as substrate [27]. All enzyme activities were expressed as U g^−1^ FW, with three biological replicates per treatment.

### 2.4. Real-Time Quantitative PCR (RT-qPCR)

Total RNA was extracted from frozen pitaya peel tissues using the CTAB method, and its quality and purity were assessed using a NanoPhotometer N50 (Implen, Munich, Germany). First-strand cDNA was synthesized from 1 μg of total RNA, and qRT-PCR was performed using the HiScript II One Step qRT-PCR SYBR Green Kit (Nanjing Novezan Biotechnology Co., Ltd., Nanjing, China). The UBQ gene served as an internal reference, and relative expression levels were calculated using the 2^−ΔΔCt^ method [31]. The primers are listed in Table 1. Data were visualized using GraphPad Prism 8 software.

### 2.5. Gene Cloning and Sequence Analysis

Genomic DNA was extracted from pitaya using the CTAB method, and the sequences of HuTGA and HuNPR genes were obtained from the pitaya genome database (http://www.pitayagenomic.com/) (accessed on 22 December 2025). Various bioinformatics analyses were performed, including cis-acting element analysis, multiple sequence alignment, phylogenetic tree construction, and motif analysis, using databases and tools such as PlantCARE: https://bioinformatics.psb.ugent.be/webtools/plantcare/html/ (accessed on 21 March 2025), ClustalW: http://www.genome.jp/tools-bin/clustalw (accessed on 25 March 2025), MEGA 11.0, and MEME Suite: https://meme-suite.org/meme/tools/meme (accessed on 26 March 2025).

### 2.6. Subcellular Localization of HuTGA1

The full-length coding sequence of *HuTGA1* was PCR-amplified and cloned into the pCAMBIA1302 vector to generate a 35S: HuTGA1-GFP fusion construct. This construct, along with a 35S: GFP control vector, was introduced into *Agrobacterium tumefaciens* GV3101(TOLOBIO, Biotech Co., Ltd., Nanjing, China) and transiently expressed in tobacco cells. GFP fluorescence was then examined under a fluorescence microscope, and nuclear localization was confirmed using DAPI (Real Times Biotechnology, Beijing, China) staining as a reference marker.

### 2.7. Yeast One-Hybrid Assay (Y1H)

Y1H was conducted using the Matchmaker Gold Y1H System to investigate the interaction between HuTGA1 and the promoters of *HuNPR1* and *HuNPR5-1*. The promoters were cloned upstream of the HIS reporter gene in the pHIS2 vector, generating pHIS2-HuNPR1pro and pHIS2-HuNPR5-1pro constructs. *HuTGA1* was inserted into the pGADT7 vector to generate pGADT7-HuTGA1 [31]. Self-activation of the promoter constructs was assessed in yeast strain Y187(TOLOBIO, Biotech Co., Ltd., China), and interaction assays were performed by co-transforming Y187 cells with pGADT7-HuTGA1 and either pHIS2-HuNPR1pro or pHIS2-HuNPR5-1pro. Positive clones were selected on SD/-Trp/-Leu plates and subsequently evaluated for protein-DNA interactions on SD/-Trp/-Leu/-His medium containing 3-amino-1,2,4-triazole (3-AT). Positive and negative controls were included to validate the assay system, ensuring the reliability of the results.

### 2.8. Dual-Luciferase Reporter Assay

Dual-luciferase reporter assays were conducted to assess the transcriptional activation of *HuNPR1* and *HuNPR5-1* promoters by HuTGA1. The effector construct was generated by cloning the full-length CDS of *HuTGA1* into the pGreen II 62-SK vector, while the reporter plasmids were produced by inserting the promoter regions of *HuNPR1* and *HuNPR5-1* into the pGreen II 0800-LUC vector [31]. *Agrobacterium tumefaciens* strain GV3101 was co-transformed with the effector and reporter plasmids and co-infiltrated into *Nicotiana benthamiana* leaves. Firefly and Renilla luciferase activities were measured 48 h post-infiltration using the Dual-Luciferase Reporter Assay System, and relative luciferase activity was calculated as the ratio of firefly to Renilla signals. This assay enabled the evaluation of *HuTGA1*’s ability to transcriptionally activate the *HuNPR1* and *HuNPR5-1* promoters.

### 2.9. Transient Overexpression of HuTGA1 in Pitaya Fruit

The full-length coding sequence of *HuTGA1* was cloned into the pGreen II 62-SK vector to generate the SK-HuTGA1 construct, which was then transformed into *Agrobacterium tumefaciens* strain GV3101 [31]. An empty pGreen II 62-SK vector served as the negative control. Agrobacterial suspensions were adjusted to an OD600 of 0.8 and injected into the pericarp of pitaya fruit. The fruit was then incubated in darkness at 25 ± 1 °C with 90–95% relative humidity, and samples were harvested at 0, 4, and 8 d post-infiltration (dpi) for subsequent analyses. Each treatment consisted of three biological replicates, with four fruits per replicate, allowing for the assessment of *HuTGA1*’s role in pitaya fruit through transient overexpression.

### 2.10. Statistical Analysis

All experiments were conducted with three independent biological replicates. Data were analyzed using SPSS version 25.0 and subjected to one-way ANOVA at a significance level of *p* < 0.05. Pairwise comparisons between treatments were assessed using Student’s *t*-test. Normality and homogeneity of variances were verified using the Shapiro–Wilk and Levene’s tests, respectively. Correlation analyses were performed using OriginPro 2021, and graphs were generated using GraphPad Prism 9.5. Significance levels are indicated as (* *p* < 0.05, and ** *p* < 0.01).

## 3. Results

### 3.1. Vanillin Treatment Enhances Disease Resistance and Maintains the Quality of Pitaya Fruit During Storage

**Figure 1:**
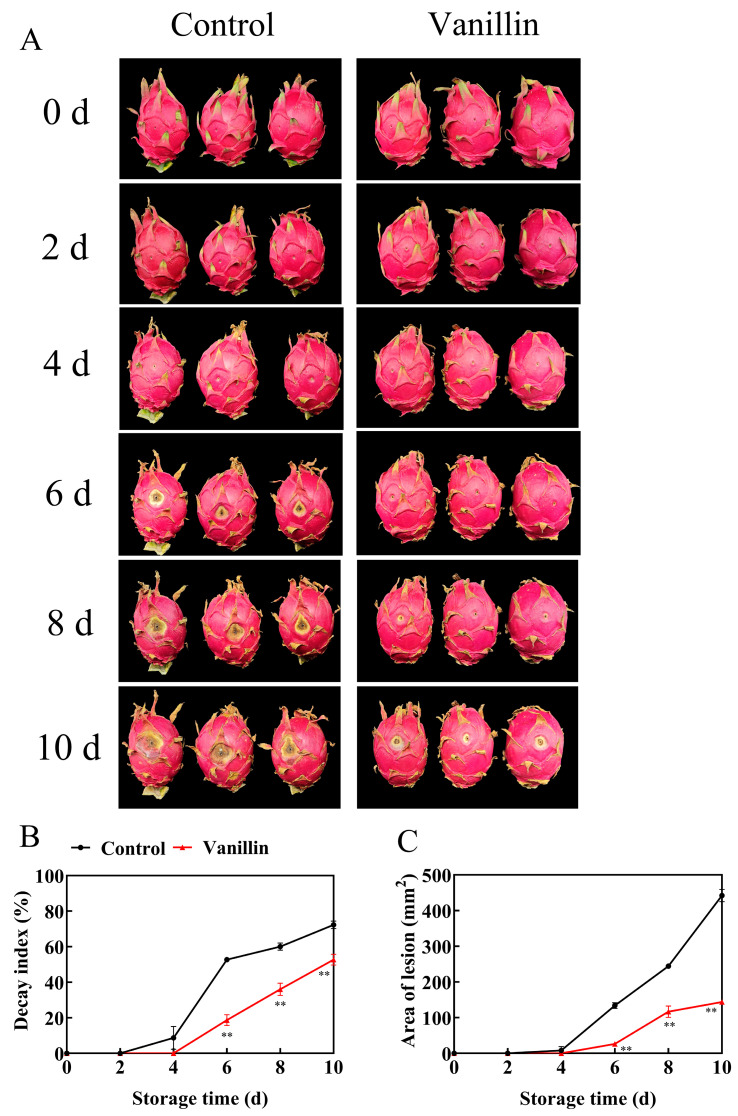
Effects of vanillin treatment on pathogenicity during pitaya storage. Changes in fruit appearance (**A**), decay index (**B**) and area of lesion (**C**) of pitaya fruit after inoculation at 25 °C. Vertical bars represent the standard error of the mean, and the asterisks indicate a significant difference between the two groups at the corresponding sampling point (** *p* < 0.01).

To evaluate the effects of vanillin treatment on the postharvest quality of pitaya, this study systematically monitored the physicochemical changes of fruits during storage (Figure 1A). Vanillin curtailed disease from 4 d onward: by 10 d, the decay index and lesion area were lower than the control by 27.12% and 67.43%, respectively (Figure 1B,C, *p* < 0.01).

**Figure 2:**
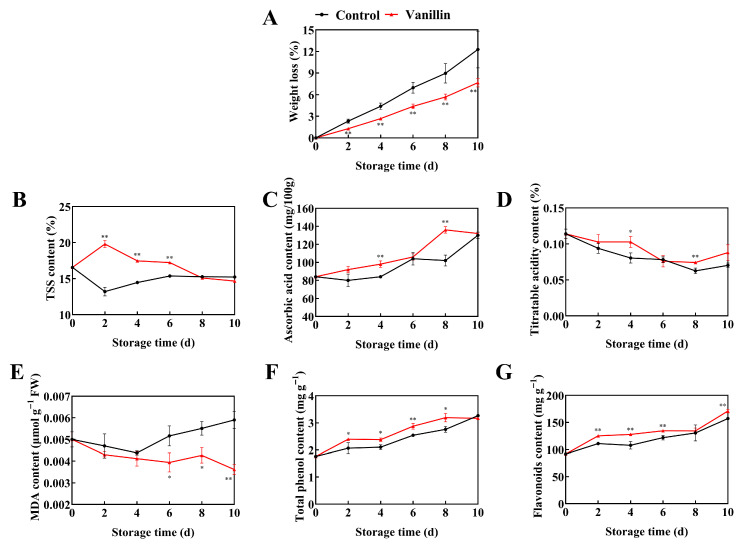
Effects of vanillin treatment on quality attributes of pitaya fruit during storage. Changes in weight loss (**A**) and contents of total soluble solids (TSS) (**B**), ascorbic acid (**C**), titratable acidity (**D**), malondialdehyde (MDA) (**E**), total phenolics (**F**) and flavonoids (**G**) of pitaya fruit during storage at 25 °C. Vertical bars represent the standard error of the mean, and the asterisks indicate a significant difference between the two groups at the corresponding sampling point (* *p* < 0.05, ** *p* < 0.01).

Weight loss increased over time in both groups but rose more slowly with vanillin; at 8 and 10 d it was reduced by 36.68% and 37.54% versus control, respectively (Figure 2A, *p* < 0.01). Furthermore, vanillin treatment promoted the accumulation of TSS, with a peak content of 19.8% on 2 d, 1.5-fold the control (Figure 2B, *p* < 0.01). The AsA accumulated throughout storage and was higher with vanillin by 16.66% on 4 d and 33.34% on 8 d, respectively (Figure 2C, *p* < 0.01). Additionally, although TA declined overall, vanillin treatment attenuated this decrease, yielding 25% and 16.67% higher TA than the control on 4 d and 8 d, respectively (Figure 2D, *p* < 0.05). The control group showing 1.3 and 1.29-fold significantly higher MDA content on 6 d and 8 d, respectively, compared to the vanillin-treated group (Figure 2E, *p* < 0.01). Moreover, vanillin treatment increased total phenolics and flavonoids to 1.13 and 1.10-fold of the control on 6 d, respectively (Figure 2F,G, *p* < 0.01). Vanillin treatment significantly slowed quality decline during storage, reducing disease and physiological damage while preserving nutritional and secondary metabolite levels.

### 3.2. Vanillin Treatment Regulates Redox Balance and Enhances Defense Enzyme Activities in Pitaya Fruit

**Figure 3:**
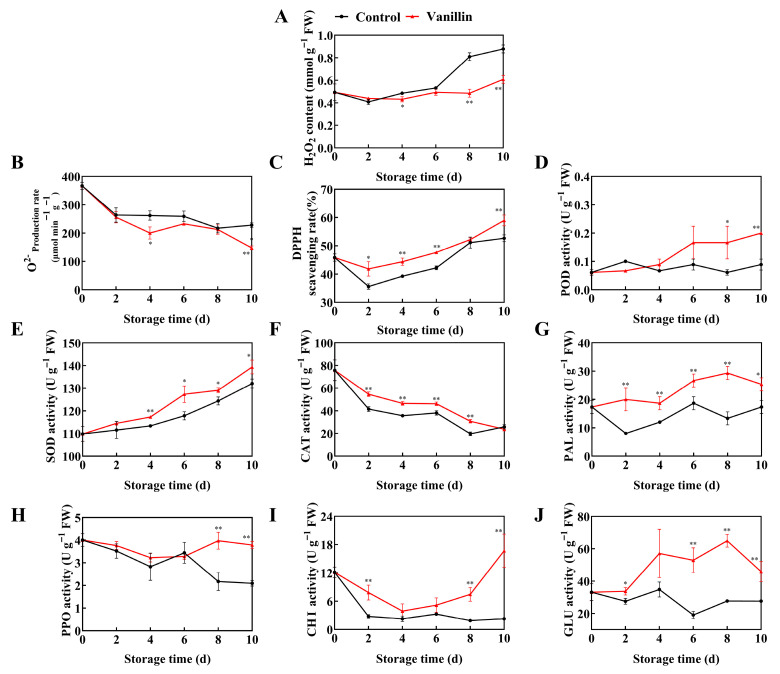
Effect of vanillin treatment on ROS levels and the activity of defense-related enzymes during pitaya fruit storage. Changes in H_2_O_2_ content (**A**), O_2_^•−^ generation rate (**B**) and DPPH scavenging rate (**C**), activities of peroxidase (POD) (**D**), superoxide dismutase (SOD) (**E**), catalase (CAT) (**F**), phenylalanine ammonia-lyase (PAL) (**G**), polyphenol oxidase (PPO) (**H**), chitinase (CHI) (**I**) and *β*-1,3-glucanase (GLU) activity (**J**) in pitaya fruit during storage at 25 °C. Vertical bars represent the standard error of the mean, and the asterisks indicate a significant difference between two groups at the corresponding sampling point (* *p* < 0.05, ** *p* < 0.01).

Our results demonstrated that vanillin treatment significantly affected the redox balance and defense enzyme system in pitaya fruit during storage. The H_2_O_2_ content increased continuously, but accumulation was markedly lower in the vanillin treatment group; by 10 d it reached 0.88 mmol g^−1^ FW in the control and 0.61 mmol g^−1^ FW in the vanillin treatment (Figure 3A, *p* < 0.01). Similarly, the production rate of O_2_^•−^ declined in both groups, yet the vanillin treatment consistently maintained lower levels, being 1.54-fold lower than the control at 10 d (Figure 3B, *p* < 0.01). In contrast, DPPH radical scavenging activity increased throughout storage, with vanillin treatment maintaining higher values—1.13, 1.13, and 1.12-fold higher than the control at 4, 6, and 10 d, respectively (Figure 3C, *p* < 0.01).

Furthermore, vanillin treatment also influenced antioxidant enzymes. POD activity increased gradually in the treatment group but remained stable in the control; at 8 and 10 d it was 2.83 and 2.22-fold higher, respectively (Figure 3D, *p* < 0.01). SOD activity rose in both groups but was consistently higher under vanillin treatment, showing 7.47%, 3.65%, and 5.26% greater activity from 6 to 10 d (Figure 3E, *p* < 0.01). CAT activity declined overall but decreased more slowly with vanillin treatment; from 2 to 8 d it was 1.32, 1.31, 1.21 and 1.56-fold higher than in the control (Figure 3F, *p* < 0.01). Defense-related enzymes were also activated. PAL activity increased and peaked at 8 d at a level 2.2-fold higher than the control (Figure 3G, *p* < 0.01). PPO activity fluctuated, with the vanillin-treated group being 45.34% and 44.73% significantly higher than the control group on 8 d and 10 d, respectively (Figure 3H, *p* < 0.01). CHI activity first decreased then rose, reaching 16.69 U g^−1^ at 10 d, 7.37-fold higher than the control (Figure 3I, *p* < 0.01). GLU activity was likewise enhanced, peaking at 8 d at 2.34-fold higher than the control (Figure 3J, *p* < 0.01). Thus, Vanillin treatment enhanced antioxidant and defense enzyme activities, improved the resistance and disease tolerance of the pitaya fruit.

### 3.3. The Expression Patterns of Defense-Related Genes in Pitaya Treated with Vanillin

**Figure 4:**
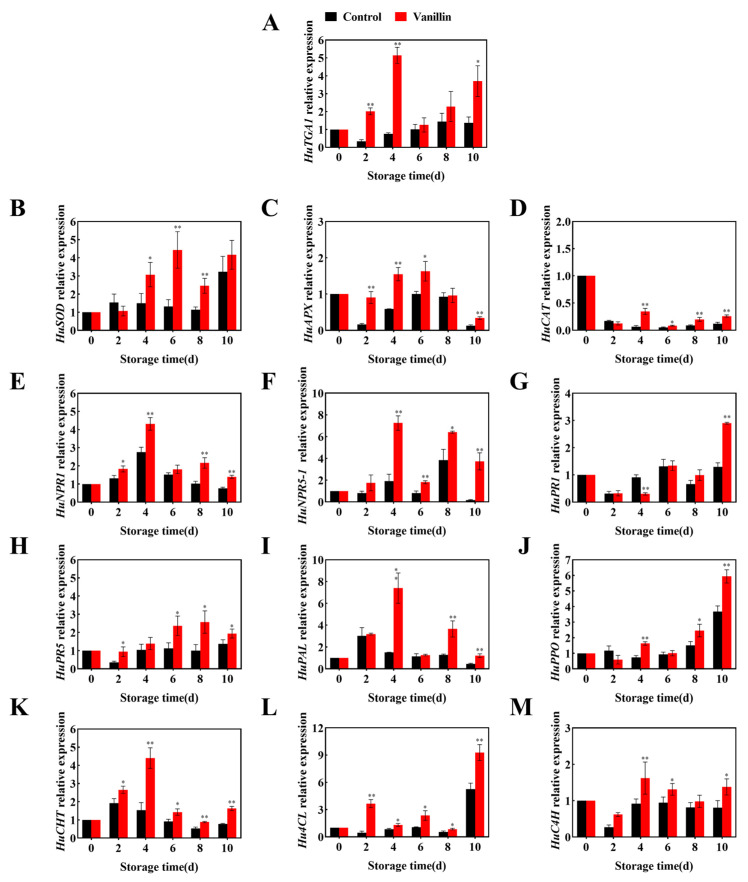
Effect of vanillin treatment on disease resistance gene expression during pitaya fruit storage. Relative expressions of *HuTGA1* (**A**), *HuSOD* (**B**), *HuAPX* (**C**), *HuCAT* (**D**), *HuNPR1* (**E**), *HuNPR5-1* (**F**), *HuPR1* (**G**), *HuPR5* (**H**), *HuPAL* (**I**), *HuPPO* (**J**), *HuCHI* (**K**), *Hu4CL* (**L**) and *HuC4H* (**M**) in pitaya fruit during storage at 25 °C. Vertical bars represent the standard error of the mean, and the asterisks indicate a significant difference between the two groups at the corresponding sampling point (* *p* < 0.05, ** *p* < 0.01).

Vanillin treatment significantly affected the expression of antioxidant- and defense-related genes in pitaya fruit during storage. Overall, gene expression levels were higher under vanillin treatment than in the control, with most showing an upregulation trend over time. Antioxidant genes (*HuSOD*, *HuAPX*, *HuCAT*) were strongly induced. *HuSOD* expression peaked at 3.36-fold the control on 6 d and remained elevated thereafter (Figure 4B, *p* < 0.01). *HuAPX* reached 1.63-fold at 6 d before slightly declining (Figure 4C, *p* < 0.05). *HuCAT* exhibited a decline–increase pattern but maintained higher expression from 6 to 10 d, reaching 2.17-fold the control on 10 d (Figure 4D, *p* < 0.01). Vanillin treatment also promoted early activation of defense signaling genes (*HuTGA1*, *HuNPR1*, *HuNPR5-1*), which peaked at 4 d and were 6.78, 1.57, and 3.8-fold higher than the control, respectively (Figure 4A,E,F, *p* < 0.01). Pathogenesis-related genes (*HuPR1*, *HuPR5*) remained upregulated throughout storage; *HuPR1* reached its maximum on 10 d (55.17% higher), while *HuPR5* peaked on 8 d (61.09% higher) (Figure 4G,H, *p* < 0.05). Furthermore, vanillin treatment enhanced secondary metabolism-related genes (*HuPAL*, *HuPPO*, *HuCHI*). On 4 d, *HuCHI* and *HuPAL* were 2.93 and 4.93-fold higher than the control, and *HuPPO* was 1.61-fold higher on 10 d (Figure 4I–K, *p* < 0.01). Hu4CL and HuC4H were also induced, peaking on 4 and 10 d, respectively, both about 43.21% above the control (Figure 4L,M, *p* < 0.01). Overall, vanillin treatment upregulated multiple antioxidant (*HuSOD*, *HuCAT*, *HuAPX*) and defense-related (*HuNPR1*, *HuPR5*, *HuPAL*, *HuCHI*) genes, strengthening fruit antioxidant capacity and resistance, thus delaying quality deterioration.

### 3.4. Correlation Analysis Between Postharvest Disease Resistance, Antioxidant Traits, and TGA-NPR Interaction

**Figure 5:**
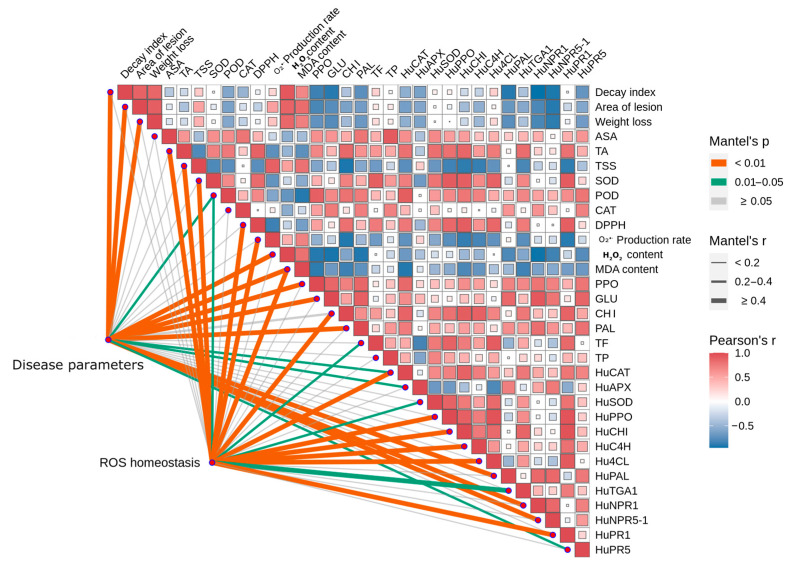
Correlation analysis between decay index, lesion area, ROS accumulation, enzyme activities and gene expressions.

Pearson correlation analysis was conducted to explore the relationship between postharvest resistance, antioxidant traits, and the TGA–NPR pathway (Figure 5). Significant positive correlations were observed among most antioxidant and defense-related parameters, especially between *HuTGA1* and NPR signaling genes. *HuTGA1* expression was positively correlated with *HuNPR1*, *HuNPR5-1*, and the downstream gene *HuPR1*, suggesting coordinated regulation of defense responses. It was also positively associated with phenylpropanoid pathway genes (*HuPAL*, *HuC4H*), implying a role in secondary metabolite synthesis. Antioxidant genes (*HuSOD*, *HuCAT*) were positively correlated with SOD and POD enzyme activities, indicating transcriptional control of enhanced antioxidant capacity under vanillin treatment. Similarly, defense-related enzyme activities (PAL, PPO, CHI) were positively correlated with *HuTGA1* expression (*p* < 0.05). These correlation analyses revealed a close association of *HuTGA1* with multiple antioxidant and defense-related traits, supporting its functional role as a key regulatory factor in postharvest resistance. The results provide insight into the molecular mechanisms underlying the enhanced disease resistance and antioxidant capacity of pitaya fruit treated with vanillin.

### 3.5. Phylogenetic, Motif, and Subcellular Localization Analysis of the HuTGA1 Gene

**Figure 6:**
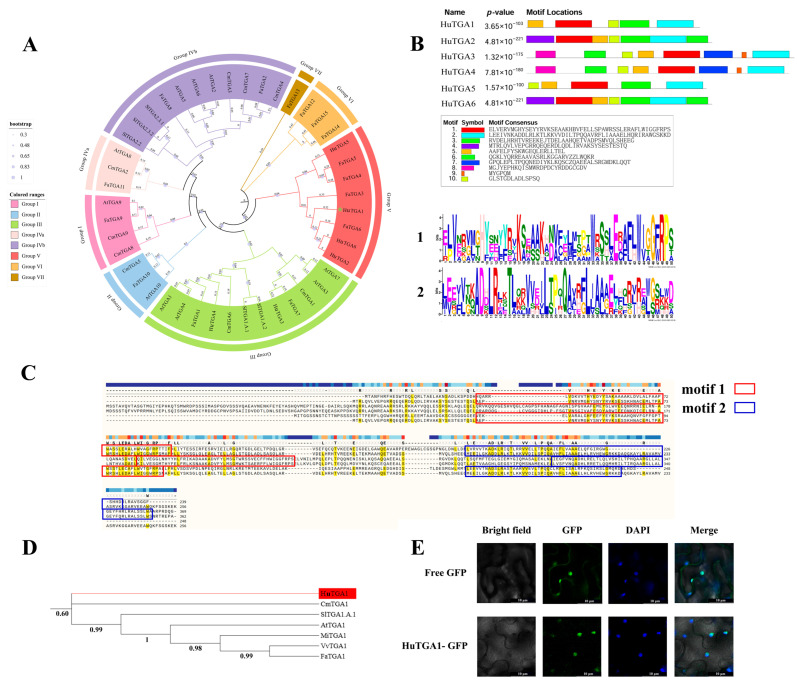
Phylogenetic, motif, and subcellular localization analyses of TGA transcription factors in pitaya. Phylogenetic tree of TGA transcription factors from Hylocereus undulatus britt and Arabidopsis thaliana HuTGA1 are highlighted in star (**A**). Motif analysis of TGA family transcription factors, showing the distribution and significance (*p*-value) of conserved motifs (Motifs 1 and 2) (**B**). Sequence logos represent the conserved amino acid sequences of each motif. Multiple sequence alignment of HuTGA family proteins, highlighting conserved motifs, and the identical amino acid regions are highlighted in yellow (**C**). Phylogenetic tree showing the relationship of HuTGA transcription factors with homologs from other species, highlighting the evolutionary placement of HuTGA1 (**D**). Subcellular localization of GFP-tagged HuTGA1 in tobacco epidermal cells. GFP fluorescence indicates nuclear localization (**E**). Scale bars = 10 μm.

*HuTGA1* was selected for in-depth characterization due to its pronounced differential expression, suggesting a potential involvement in the regulation of fruit quality and disease resistance. Phylogenetic analysis demonstrated that *HuTGA1* shares a high degree of sequence similarity with TGA transcription factors from *Arabidopsis thaliana*, melon, and strawberry (Figure 6A,D). Motif analysis further revealed that several members of the HuTGA family, including *HuTGA1*, contain multiple conserved domains critical for DNA binding and transcriptional regulation (Figure 6B). Consistent with these findings, amino acid sequence alignment confirmed the conservation of these motifs, underscoring their potential functional significance (Figure 6C). Subcellular localization analysis was performed to further elucidate the function of *HuTGA1*. The control vector (35S::GFP) exhibited green fluorescence distributed throughout both the cytoplasm and nucleus of tobacco leaf cells, whereas the HuTGA1–GFP fusion protein displayed fluorescence exclusively within the nucleus (Figure 6E). These observations indicate that HuTGA1 is a nuclear-localized protein, consistent with the characteristic localization pattern of transcription factors.

### 3.6. HuTGA1 Positively Regulates HuNPR Genes to Enhance Disease Resistance in Pitaya Fruit

**Figure 7:**
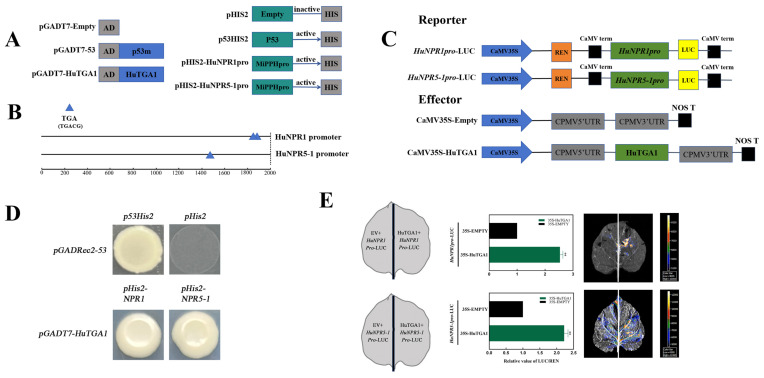
Functional analysis of HuTGA1 in regulating *HuNPR1* and *HuNPR5-1* expression. Constructs used in the yeast one-hybrid assay (**A**). Schematic representation of the *HuNPR1* and *HuNPR5-1* promoter, showing TGA-binding sites (**B**). Schematic representation of the dual-luciferase reporter assay constructs used to assess the transactivation of the *HuNPR1* and *HuNPR5-1* promoters by HuTGA1 (**C**). Y1H assay for the interaction of HuTGA1 with the as-1 motif and with promoters of *HuNPR1* and *HuNPR5-1* (**D**). Activation ability of HuTGA1 in regulating *HuNPR1* and *HuNPR5-1* (**E**). Vertical bars represent the standard error of the mean, and the asterisks indicate a significant difference between two groups at the corresponding sampling point (** *p* < 0.01).

To test whether *HuNPR* genes are direct transcriptional targets of *HuTGA1*, cis-element prediction identified two and one putative TGA-binding motifs in the promoters of *HuNPR1* and *HuNPR5-1*, respectively (Figure 7B), suggesting potential direct binding. Y1H assays confirmed this interaction: co-transformation of pHis-*HuNPR5*-*1*pro + HuTGA1-AD enabled growth on SD-His/Trp/Leu + 3-AT^100^ medium, while pHis-HuNPR1pro + HuTGA1-AD grew on SD-His/Trp/Leu + 3-AT^210^ (Figure 7A,D). No growth occurred in negative controls, verifying the specificity of the interaction.

Dual-luciferase reporter (DLR) assays in *Nicotiana benthamiana* leaves further supported transcriptional activation. Co-expression of *HuTGA1* with HuNPR1pro or HuNPR5-1pro enhanced LUC/REN ratios by 2.53 and 2.22-fold, respectively (Figure 7C,E), confirming that HuTGA1 functions as a positive regulator of *HuNPR1* and *HuNPR5-1* expression.

**Figure 8:**
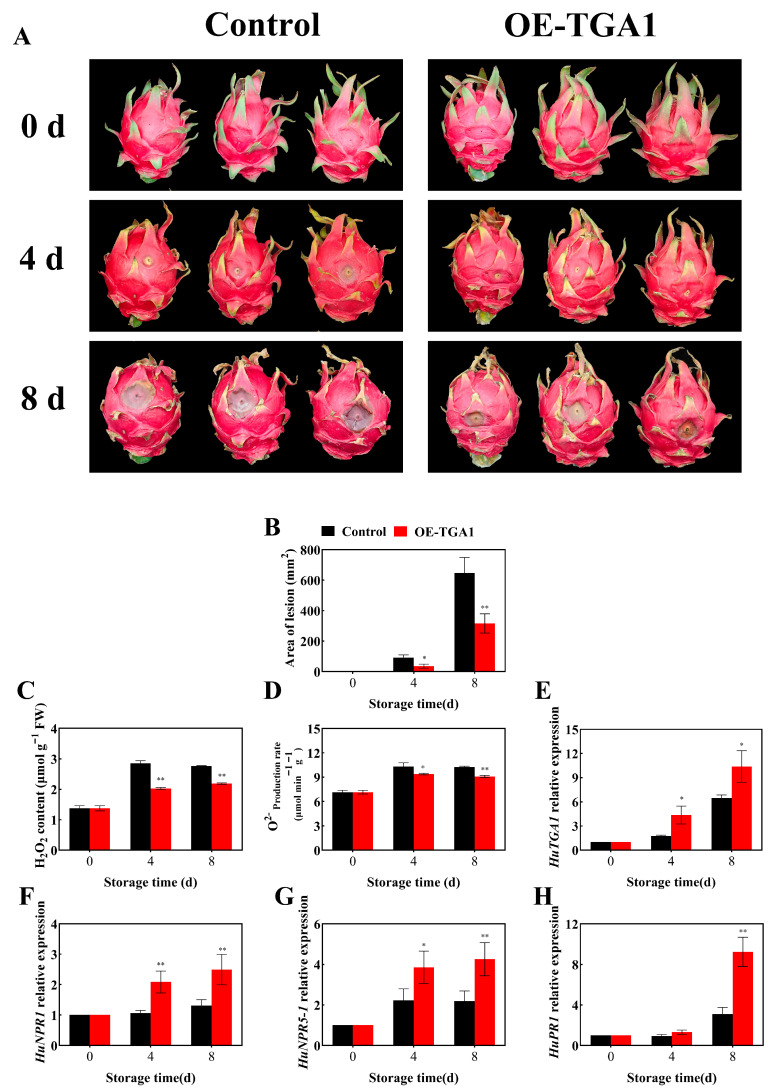
Transient overexpression of *HuTGA1* in pitaya fruit. Phenotypic diagram of pitaya after infection (**A**). Changes in area of lesion (**B**), H_2_O_2_ content (**C**) and O_2_^•−^ generation rate (**D**), relative expressions of *HuTGA1* (**E**), *HuNPR1* (**F**), *HuNPR5-1* (**G**) and *HuPR1* (**H**) in pitaya fruit during storage at 25 °C. Vertical bars represent the standard error of the mean, and the asterisks indicate a significant difference between the two groups at the corresponding sampling point (* *p* < 0.05, ** *p* < 0.01).

Functional overexpression (*OE-TGA1*) in pitaya further validated its role. Compared with the control, *OE-TGA1* fruits exhibited reduced lesion areas by 60.46% and 51.21% at 4 and 8 d post-inoculation, respectively (Figure 8B, *p* < 0.05). ROS analysis showed that H_2_O_2_ contents in OE-TGA1 fruits were 0.71- and 0.79-fold of those in the control at 4 d and 8 d, respectively, while O_2_^•−^ production rates decreased by 8.93% and 11.13%, respectively (Figure 8C,D, *p* < 0.05). Expression profiling revealed that *HuTGA1* overexpression upregulated key defense genes: *HuTGA1* transcripts were 2.47 and 1.61-fold higher than the control at 4 and 8 d; *HuNPR1* and *HuNPR5-1* increased 1.89 and 1.95-fold, and *HuPR1* reached 2.97-fold at 8 d (Figure 8E–H, *p* < 0.01).

## 4. Discussion

Developing safe, effective methods to control postharvest diseases like *F. oxysporum*-induced soft rot remains a key challenge in pitaya research. Our study shows that vanillin treatment significantly inhibits this disease, reducing disease index, lesion area and weight loss, while improving fruit firmness, titratable acidity, soluble solids, ascorbic acid content and DPPH radical scavenging activity. These findings are consistent with previous studies in tomato and citrus fruits [32,33]. Moreover, previous studies have demonstrated that vanillin exhibits synergistic effects when combined with other natural compounds, such as chitosan or taurine, enhancing its preservative and antioxidant properties [33,34]. In addition, the transient increase in TSS observed in the control treatment is most likely due to dehydration-induced concentration effects, followed by senescence-associated and pathogen colonization consumption processes.

Vanillin’s preservative mechanisms involve activating antioxidant systems and pathogenesis-related enzymatic pathways. Our results show that vanillin treatment elevates the activities of antioxidant enzymes (SOD, POD, CAT), thus maintaining ROS homeostasis. Enhanced ROS scavenging alleviated lipid peroxidation, as confirmed by reduced MDA accumulation. Amid oxidative stress and pathogen invasion, sustained high enzyme activities preserved fruit quality and inhibited pathogen growth. These results are consistent with previous studies [10,13]. Evidence also indicates that fruit defense responses to biotic/abiotic stress involve a complex interplay of antioxidant regulation, dynamic cellular changes, and associated defense mechanisms. At low concentrations, ROS can stimulate pathogenesis-related protein synthesis and promote cell wall cross-linking and lignification, thereby enhancing fungal resistance. However, excessive ROS accumulation—beyond scavenging capacity—induces oxidative damage, accelerates senescence, and impairs fruit quality and disease resistance [6,35]. Furthermore, SOD catalyzes the conversion of O_2_^•−^ to H_2_O_2_, which is further decomposed into water by CAT and POD, thus mitigating oxidative stress. Moreover, coordinated PPO and POD activity promotes polyphenol polymerization into lignin and the formation of hydroxyproline-rich glycoproteins (HRGPs), creating a structural barrier that enhances fruit resistance to invading pathogens [22,36]. Multiple studies have demonstrated that exogenous treatments can effectively activate the antioxidant system, thereby delaying senescence and enhancing disease resistance in fruits. For instance, Du et al. [37] reported that Artemisia annua extract significantly increased the activities of SOD, POD, and CAT in pepper, while reducing H_2_O_2_ and MDA levels, thereby mitigating oxidative damage. Similarly, glycine betaine (GB) treatment elevated the activities of antioxidant enzymes, including SOD, CAT, APX, and POD, reducing ROS accumulation and preserving postharvest blueberry quality [38]. In litchi, application of a vanillin–taurine Schiff base (VTSB) not only delayed pericarp browning and weight loss but also lowered MDA content and electrolyte leakage, while maintaining higher levels of anthocyanins, TP, and antioxidant enzyme activities [39].

Moreover, vanillin treatment also activates the phenylpropanoid pathway, which was supported by the increased biosynthesis of antimicrobial compounds such as TP and TF [4,40]. Additionally, vanillin-induced upregulation of PPO and POD activities further promotes TP and TF oxidation and polymerization, enhancing lignin deposition and phenolic cross-linking to restrict pathogen penetration and slow lesion expansion. GLU and CHI hydrolyze major components of fungal cell walls, playing essential roles in defending against pathogen invasion [4,41]. Previous studies have shown that increased PAL, GLU, and CHI activities, along with the accumulation of phenolics and flavonoids, effectively enhance blueberry resistance to *Botrytis cinerea* [41]. This study shows that vanillin treatment significantly increases the activities of antioxidant enzymes (SOD, POD, CAT) and defense-related enzymes (PPO, GLU, PAL, CHI), along with the expression of their corresponding genes. Moreover, vanillin markedly promotes the accumulation of secondary metabolites (total phenolics, flavonoids), further boosting fruit antioxidant and defense capacity. These findings indicate that vanillin mitigates ROS accumulation and enhances pitaya resistance to pathogens by activating antioxidant and pathogenesis-related enzymatic pathways, thereby reducing postharvest soft rot incidence. Similarly, Chen et al. [42] reported that GABA treatment significantly upregulated the expression of phenylpropanoid metabolism-related genes (PAL, C4H, 4CL) and their enzymatic activities, while also boosting POD and PPO activities, thereby strengthening mango resistance to *Colletotrichum gloeosporioides*.

**Figure 9:**
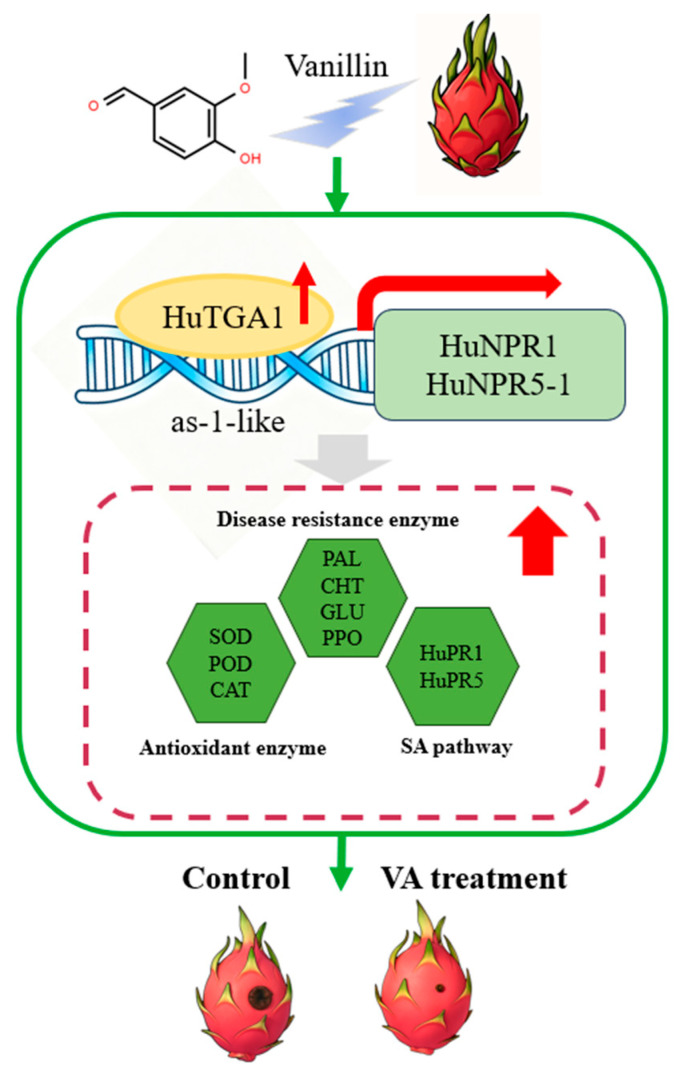
Proposed model illustrating the regulatory role of *HuTGA1* in VA-induced disease resistance against *F. oxysporum* in pitaya fruit.

SA is a key mediator of plant immune responses, inducing systemic acquired resistance (SAR) and boosting disease resistance. NPR1, the primary SA receptor, is indispensable for SAR activation. TGA transcription factors—core regulators of the SA signaling cascade—coordinate plant stress and immune responses. Studies on kiwifruit and strawberry show that TGA genes respond to hormonal signals and pathogen infection, and their overexpression enhances disease resistance [39]. These findings highlight the critical role of TGA transcriptional activation in enhancing plant disease resistance. Our results show that vanillin treatment upregulates SA signaling-related defense genes (*HuTGA1*, *HuNPR1*, *HuNPR5-1*). *HuTGA1*, a nuclear-localized transcriptional activator, directly binds to and activates the promoters of *HuNPR1* and *HuNPR5-1*, establishing a regulatory link between *HuTGA1* and NPR genes. This mechanism aligns with previous studies demonstrating TGA transcription factors’ crucial role in plant immune response regulation [43,44]. Overall, our findings strongly demonstrate that vanillin enhances pitaya’s disease resistance and antioxidant capacity by activating the HuTGA1-NPR module, which regulates defense gene expression and activates antioxidant and pathogenesis-related enzymatic pathways (Figure 9). These results provide valuable insights for developing safe, effective strategies to control pitaya postharvest diseases.

## 5. Conclusions

In conclusion, vanillin treatment reduces soft rot and delays postharvest deterioration in pitaya by boosting antioxidant capacity, activating defense-related enzymes, and accumulating phenolics and flavonoids. HuTGA1, a vanillin-inducible transcriptional activator, regulates fruit defense responses by activating *HuNPR1* and *HuNPR5-1*; its overexpression enhances ROS scavenging and upregulates defense genes. These findings indicate that vanillin enhances pitaya systemic resistance via the HuTGA1-HuNPR signaling module. Future research should focus on developing combined vanillin-based preservation systems with other substances or technologies and extending their application to postharvest storage of diverse fruits including pitaya.

## Figures and Tables

**Table 1:** Sequence of primers for the RT-qPCR gene of pitaya.

Gene	Forward Primer (5′-3′)	Reverse Primer (5′-3′)
Nonexpressor of Pathogenesis-Related genes 1 (*HuNPR1*)	GACAGACGAAAGGAGCTTGG	CCACAGCATAGTGGAGAGCA
Pathogenesis-Related protein 1 (*HuPR1*)	GCTCGAGCTTCCCCTAGTTT	GCCCAAAGCTTAACAGCATC
Pathogenesis-Related protein 5 (*HuPR5*)	GCGGATACACTCCACCAAGT	TGCAGGGAAGGGTAAGAGTG
TGACG motif-binding factor 1 (*HuTGA1*)	CTACGAGGACTACTACAGCG	TAGATGAGGTGGAAGATGG
Nonexpressor of Pathogenesis-Related genes 5-1 (*HuNPR5-1*)	TCAAGTCTCCATCGTCCC	TCAGCACCCTCATCACAT
Polyphenol Oxidase (*HuPPO*)	TGTCAGGAGGCCAAAGAAGT	CCTGCATATTCGGCTTTGAT
Chitinase (*HuCHI*)	CAGTCCTGGTCCCAATGCTC	CTTCTGCCATTTGATCGCGG
Peroxidase (*HuPOD*)	CCATCCCAAATCGCACTATA	ATCGGTCCTCAGCATGAAAT
Phenylalanine Ammonia-Lyase (*HuPAL*)	AAGGAACTTCGGCTATCCCG	ACTCCTCCCCAGGAGACTTC
Cinnamate 4-Hydroxylase (*HuC4H*)	AAGTTGAAGCTCCCACCAGG	CTGGCCCATCCTAAGCAACA
4-Coumarate: CoA Ligase (*Hu4CL*)	TCTTCAAATCACGCCTCCCC	GTTGGAGATGAGACAGGGGC
Catalase (*HuCAT*)	TGCATCCAATACAGGGGAACT	GTTTGGCTTGAATGCGTGGA
Superoxide Dismutase (*HuSOD*)	ACGCTCCGAGAATCTATCCA	CTTTGCACGTACAGTAGGGGA
Ascorbate Peroxidase (*HuAPX*)	AAGGAACGCAACCCTTCCAA	AACCTCTGCCATGGGAAACC
Ubiquitin (*HuUBQ*)	TGAATCATCCGACACCAT	TCCTCTTCTTAGCACCACC

## Data Availability

The original contributions presented in this study are included in the article. Further inquiries can be directed to the corresponding author.
